# Establishment of clinical diagnosis model of Graves’ disease and Hashimoto’s thyroiditis

**DOI:** 10.1186/s12967-018-1765-3

**Published:** 2019-01-07

**Authors:** Zhaohui Cui, Zhixiao Wang, Xiaoyun Liu, Yun Cai, Xinyu Xu, Tao Yang

**Affiliations:** 1 Department of Endocrinology, The First Affiliated Hospital of Nanjing Medical University, Nanjing, 210029 People’s Republic of China; 2 Department of Endocrinology, The Affiliated Huaian NO.1 People’s Hospital of Nanjing Medical University, Huai’an, 223300 People’s Republic of China

**Keywords:** Graves’ disease, Hashimoto’s thyroiditis, Model, Diagnosis

## Abstract

**Background:**

A clinical diagnosis model include thyroid functions, thyroid antibodies and radioactive iodine uptake (RAIU) of patients with hyperthyroidism were established and as new evaluation indicators for the differentiation of the Graves’ disease (GD) and Hashimoto’s thyroiditis (HT).

**Methods:**

Clinical data of patients with newly diagnosed hyperthyroidism including gender, age, thyroid function, thyroid antibodies (FT3, FT4, TSH, TPOAb, TGAb, TRAb), RAIU (2 h, 6 h, 24 h) were collected. A stepwise regression analysis was performed to establish a model based on these variables.

**Results:**

Model 1 was subjected to stepwise regression analysis. After screening, the variables that entered the model included FT3, TGAb, TPOAb, TRAb, 2-h RAIU, 24-h RAIU and gender, in which the variables FT3, TGAb, TRAb, 2-h RAIU, 24-h RAIU, and gender were significantly different. Model 2 without RAIU was also subjected to stepwise regression analysis. After screening, the variables that entered the model included FT4, TGAb, TPOAb, TRAb and gender were statistical significant. The larger value of each variable in the two models indicated the higher probability to diagnose GD. The area under the receiver operating characteristic (ROC) curve of model 1 was 0.843 (95% CI 0.779–0.894), and the area under the ROC curve of model 2 was 0.806 (95% CI 0.685–0.824), which showed good differential diagnostic value.

**Conclusions:**

GD and HT diagnosis model was established according to the variables including gender, FT3, TGAb, TRAb, the 2-h RAIU, the 24-h RAIU in the model 1, and the variables FT4, TGAb, TPOAb, TRAb and gender in the model 2 that did not include RAIU. These models had high value to differentiate GD and HT for patients with early hyperthyroidism.

## Background

Graves’ disease (GD) is a common organ-specific autoimmune endocrine disease, with an occurrence of 1.2% in the Chinese population. Autoimmune thyroid disease (AITD) includes two major clinical manifestations: GD and Hashimoto’s thyroiditis HT [1]. The prevalence of AITD is approximately 5% [1, 2], and the clinical features of this disease are mainly hyperthyroidism and hypothyroidism. Although the pathogenesis is not very clear, there is evidence that environmental factors (infection, drugs, smoking, iodine, etc.) could trigger AITD in susceptible individuals [3–6]. Case reports have shown that HT can progress to GD [7–10]. In addition, other studies have reported that about 10–15% of patients with GD may have hypothyroidism after anti-thyroid treatment [11], suggesting that GD and HT may exist at the same time. In some cases, GD patients may have lymphocytic thyroiditis, and treatment with antithyroid drugs will lead to drug-induced hypothyroidism. Therefore, for patients with early hyperthyroidism, it is crucial to identify the cause of the disease.

At present, the diagnosis of GD is mainly based on the typical clinical manifestations of hyperthyroidism, diffuse enlargement (lesion) of thyroid B in ultrasound, and positive expression of thyrotropin receptor antibody (TRAb), thyroglobulin antibody (TGAb), and thyroid peroxidase antibody (TPOAb). However, in the early stage of HT, there may also be clinical manifestation of hyperthyroidism, positive TGAb and TPOAb. For example, 70% of GD patients have positive TPOAb and TGAb. Similarly, TRAb is also positive in a few HT patients [12]. Therefore, it is difficult for clinicians to distinguish GD and HT with atypical clinical symptoms and positive antibodies.

Currently, the most valuable laboratory test is the determination of serum thyroid stimulating antibody (TSAb) and thyroid stimulating blocking antibody (TSBAb). It is generally believed that TSAb is dominant in GD patients. When the dominant antibody is TSBAb, the incidence of hypothyroidism is increased [13, 14]. However, the detection methods of TSBAb and TSAb are mainly used for scientific research, and it is not applicable in clinical detection for the diagnosis and subsequent treatment of the GD and HT.

So far, there is still no effective differentiation method. Therefore, for patients with early hyperthyroidism, we hypothesize that a clinical differentiation model could be established based on thyroid function (FT3, FT4, TSH), thyroid antibodies (TGAb, TPOAb, TRAb), RAIU (2 h, 6 h, 24 h) and cytological pathology of fine needle aspiration. Model could help clinicians to quantify the indicators while making an accurate diagnosis, thus providing a reference for the clinical diagnosis and identification of hyperthyroidism to effectively save existing medical resources and reduce the economic burden on patients.

## Methods

### Research subjects

This study included 197 patients (51 males and 146 females, aged 16–68, mean age 38.30 ± 12.63) whom were admitted to the Department of Endocrinology, the First Affiliated Hospital of Nanjing Medical University, from January 2016 to September 2017. The inclusion criteria included (1) patients with initial hyperthyroidism; (2) free T3 (FT3), free T4 (FT4), and thyroid stimulating hormone (TSH) were confirmed to the initial diagnosis of hyperthyroidism; and (3) clinical data were relatively complete. Patients with hyperthyroidism during pregnancy, severe hepatic or renal dysfunction, autoimmune disease, and other types of hyperthyroidism, or patients with unsuitable conditions for participation were excluded.

### Research methods

Clinical data of patients with newly diagnosed hyperthyroidism, including gender, age, thyroid function and antibodies (FT3, FT4, TSH, TPOAb, TGAb, TRAb), and RAIU (2 h, 6 h, 24 h) were collected. Informed consents were signed by all patients. Ultrasound-guided thyroid fine needle aspiration was performed and rapid smear was done for three slides for pathological examination. The GD and HT grouping was based on the pathological findings of thyroid fine needle aspiration. FT3, FT4, TSH, TPOAb, TGAb, and TRAb levels were determined by chemiluminescence assays.

### Statistical analysis

The independent sample *t* test was used to compare the two groups of quantitative data. The quantitative data were expressed as mean ± standard deviation. Logistic regression was used for the diagnostic model equation. R software was used for all statistical analysis. The area under the RAIU curve was calculated using the trapezoidal rule. The diagnostic efficacy of the evaluation model was determined using a receiver operating characteristic (ROC) curve with α = 0.05 as the statistically significant level.

## Results

### Clinical characteristics of the study subjects

There was no significant difference between the two groups in age and TPOAb (P > 0.05). The levels of FT3, FT4 and TRAb in GD patients were significantly higher than those in HT group (P < 0.05). The level of TGAb in HT group was significantly higher than that in the GD group (P = 0.05). The RAIU at 2 h, 6 h, 24 h in the GD group was significantly higher than in the HT group. The area under the curve (AUC) for RAIU in GD group was significantly higher than that in HT group (Table 1).

**Table 1:** Clinical feature of the study subjects

Items	GD n = 119	HT n = 78	P value
Gender (M/F)	41/78	9/69	< 0.001
Age	38.73 ± 13.73	37.65 ± 10.79	0.560
FT3	20.99 ± 14.36	14.31 ± 10.91	< 0.001
FT4	52.31 ± 30.26	40.27 ± 23.88	0.003
TSH	0.01 ± 0.03	0.02 ± 0.42	0.038
TGAb	362.52 ± 587.11	550.96 ± 757.63	0.050
TPOAb	226.37 ± 209.04	274.39 ± 477.62	0.335
TRAb	9.67 ± 10.68	5.75 ± 9.6	0.010
2-h RAIU	31.15 ± 16.75	22.79 ± 17.12	< 0.001
6-h RAIU	47.92 ± 20.79	32.82 ± 23.88	< 0.001
24-h RAIU	57.74 ± 20.44	38.49 ± 26.57	< 0.001
RAIU-AUC	1109.22 ± 433.67	753.13 ± 527.79	< 0.001

M: male; F: female; GD: Graves’ disease; HT: Hashimoto’s thyroiditis; FT3: free T3; FT4: free T4; TSH: thyroid stimulating hormone; TGAb: thyroglobulin antibody; TPOAb: thyroid peroxidase antibodies; TRAb: thyrotropin receptor antibody; RAIU: radioactive iodine uptake; AUC: area under the curve

### Pathological diagnosis

**Fig. 1:**
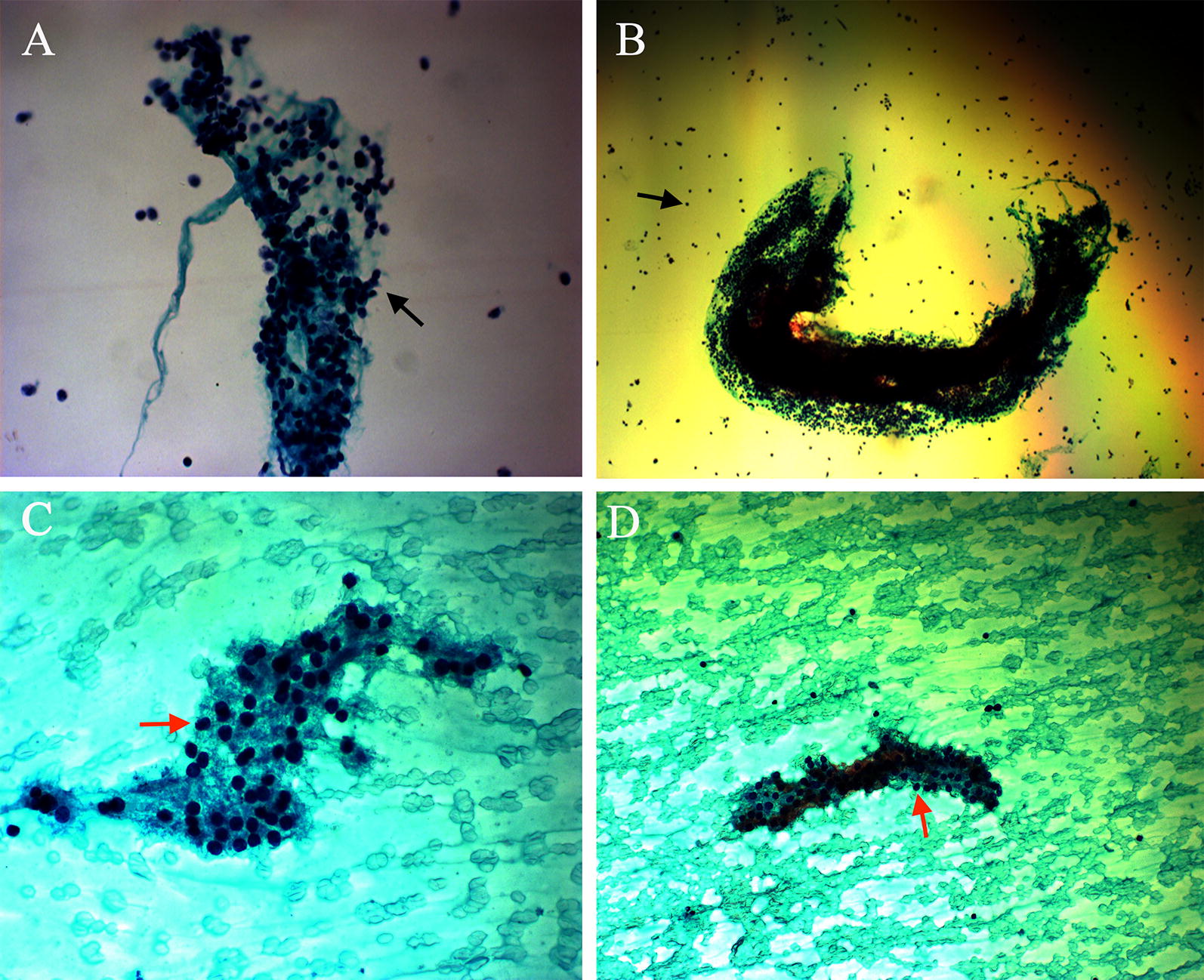
Pathological features of the two groups: **A** HT group (under light microscope, ×20); **B** HT group (under light microscope ×5); **C**, **D** GD group (under light microscope ×20). Black arrow: polymorphic lymphoid cells; Red arrow: follicular cells

Pathological results were used to diagnose GD and HT in this study. HT’s pathological diagnosis is based on frequent occurrence of polymorphic lymphoid cells (small mature lymphocytes, larger activated lymphocytes, and occasional plasma cells) and Hürthle cells, and characterized by the different ratio of these two types of cells (Fig. 1) [15]. Hyperthyroidism is diagnosed according to the pathological reports of follicular cells (single-layered, honeycomb), glia, and phagocytic cells (Fig. 1).

### Establishment of clinical diagnosis regression model

Various clinical features of the patients with hyperthyroidism were put into the model as the independent variables. Model 1 was subjected to stepwise regression analysis. After screening, the variables that entered the model included FT3, TGAb, TPOAb, TRAb, 2-h RAIU, 24-h RAIU and gender, in which the variables FT3, TGAb, TRAb, 2-h RAIU, 24-h RAIU, and gender were significantly different (P < 0.05).

Model 2 without RAIU was also subjected to stepwise regression analysis. After screening, the variables that entered the model included FT4, TGAb, TPOAb, TRAb and gender, all of which were statistically significant. The larger the value of each variable in the two models indicated the higher probability to diagnose GD. Table 2 showed the regression coefficients and risk scores for each variable. The equations for the two models are as follows:

**Table 2:** Regression coefficients and risk scores for each variable

Items	Model 1			Model 2		
	*β*	*OR*	*P*	*β*	*OR*	*P*
FT3	0.0399	1.0407	0.013935	–	–	–
FT4	–	–	–	0.0219	1.0221	0.000941
TGAb	− 0.0008	0.9992	0.004100	− 0.0012	0.9988	5.94E − 05
TPOAb	–	–	–	− 0.0011	0.9989	0.030917
TRAb	0.0709	1.0734	0.000484	0.0518	1.0532	0.005661
X2 h	− 0.0783	0.9247	0.000162	–	–	–
X24 h	0.0789	1.0821	5.04e − 08	–	–	–
Gender	− 1.9153	0.1473	0.000152	− 1.8257	0.1611	0.00013
AUC	0.843			0.806		

β: regression coefficient; OR: odds ratio; “–”: not applicable; FT3: free T3; FT4: free T4; TGAb: thyroglobulin antibody; TPOAb: thyroid peroxidase antibodies; TRAb: thyrotropin receptor antibody; X2 h: 2-h RAIU; X24 h: 24-h RAIU; AUC: area under the curve

#### Model 1

$$\begin{aligned} {\text{Logit P}} & = 0.0 3 9 9*{\text{FT3}} - 0.000 8*{\text{TGAb}} \\ & \quad + 0.0 70 9*{\text{TRAb}} - 0.0 7 8 3*{\text{X2h}} + \;0.0 7 8 9*{\text{X24h}} \\ & \quad - 1. 9 1 5 3*{\text{Gender}} + 1. 2 2 8 1\\ \end{aligned}$$

#### Model 2

$$\begin{aligned} {\text{Logit P}} & = 0.0 2 1 9*{\text{FT4}} - 0.00 1 2*{\text{TGAb}} - 0.00 1 1*{\text{TPOAb}} \\ & \quad + \;0.0 5 1 8*{\text{TRAb}} - 1. 8 2 5 7*{\text{Gender}} + 3.0 5 1 1\\ \end{aligned}$$

### Area under the curve (AUC)

**Fig. 2:**
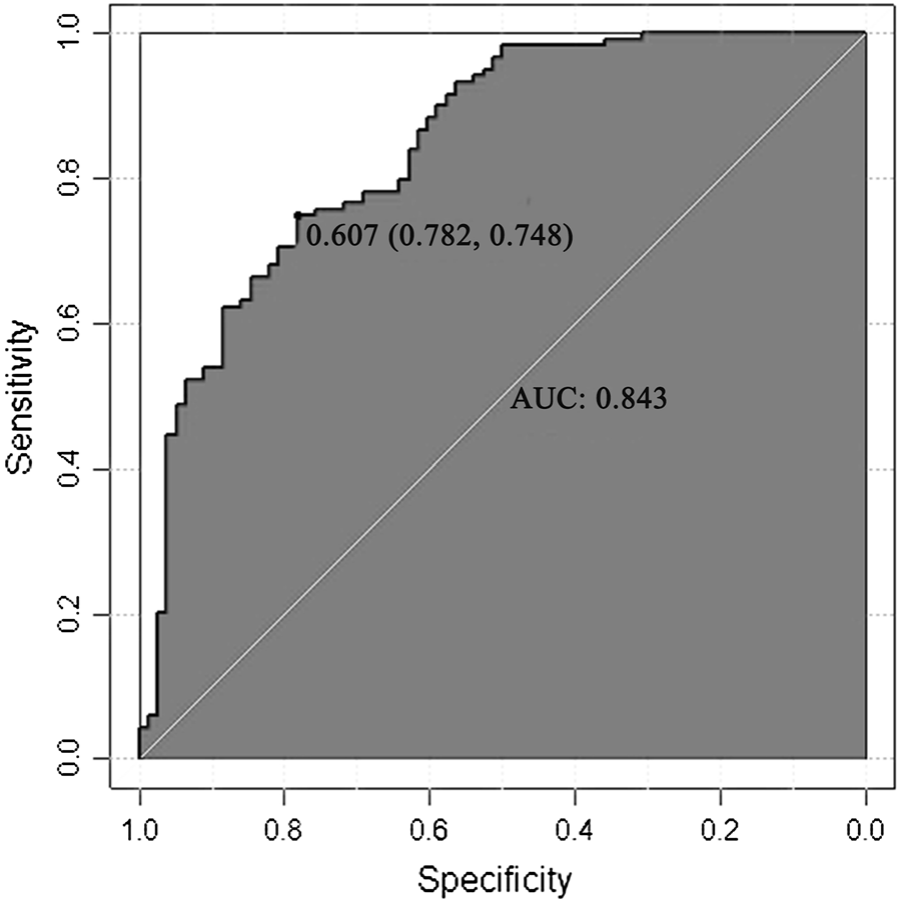
Characteristic feature of the curve for model 1

**Fig. 3:**
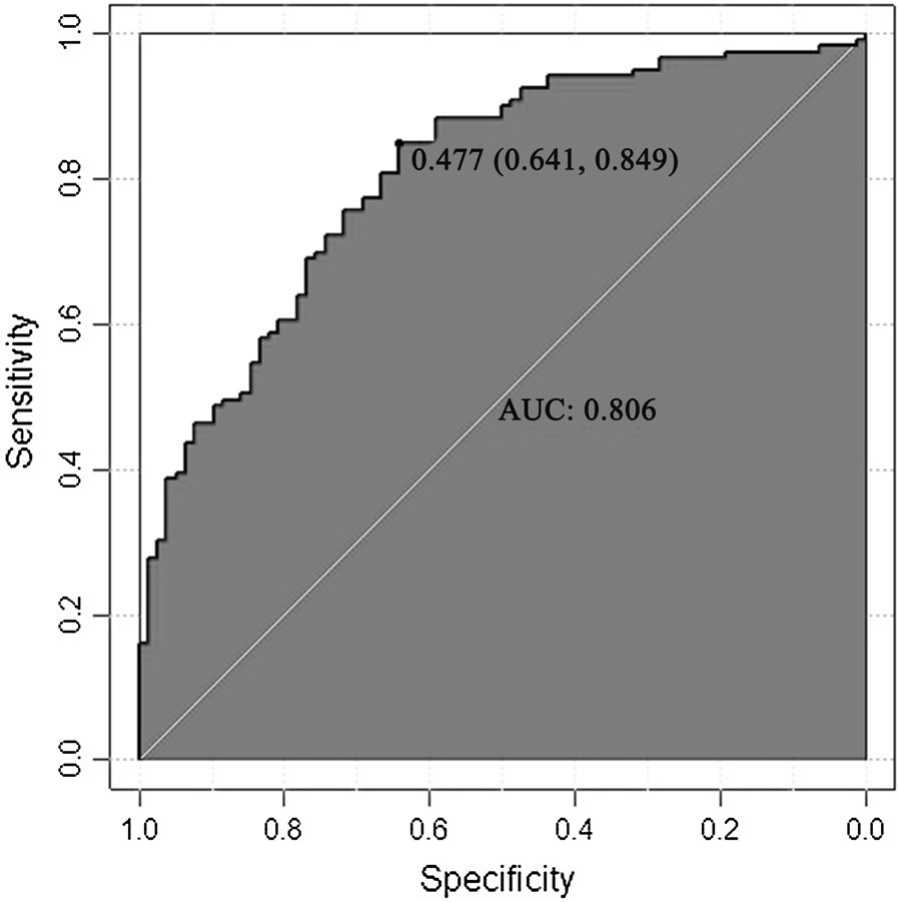
Characteristic feature of the curve for model 2

The area under the ROC curve ranged from 0.5 to 1.0. AUC < 0.7 indicated that the diagnostic accuracy was low; while AUC > 0.8 suggested that the model had a good diagnostic value. As shown in Fig. 2, AUC of model 1 was 0.843 (95% CI 0.779–0.894), and AUC of model 2 was 0.806 (95% CI 0.685–0.824) (Fig. 3), suggesting that both models had good differential diagnostic value.

### Determination of diagnostic cutoff point

The sensitivity and specificity analysis of the different diagnostic cutoff points for the two models are shown in Table 3. The maximum value of the Youden’s index is the best value for sensitivity and specificity.

**Table 3:** Sensitivity and specificity of the two models at each cutoff point

Model 1				Model 2			
Cutoff point	Sensitivity (%)	Specificity (%)	Youden’s index	Cutoff point	Sensitivity (%)	Specificity (%)	Youden’s index
0.343	43.7	95	0.386	1.888	26.1	95	0.209
− 0.389	65.5	85	0.502	1.076	51.3	85	0.359
− 0.673	75.6	75	0.512	0.437	65.5	75	0.399
− 0.596	73.1	79.5	0.526	− 0.113	78.2	62.6	0.410
− 0.981	78.2	70	0.473	0.312	67.2	70	0.377
− 1.154	83.2	65	0.472	− 0.398	87.4	50	0.374
− 1.911	96.6	50	0.466	− 0.990	95	17.9	0.129

Youden’s index = sensitivity + specificity − 1, the maximum value is the optimal value

In model 1, the sensitivity and specificity for diagnosis of GD were 73.1% and 79.5%, respectively when the cutoff point was > − 0.596. When the cutoff point was < − 0.596, HT was diagnosed.

In model 2, the sensitivity and specificity for the diagnosis of GD were 78.2% and 62.6%, respectively when the cutoff point is > − 0.113. In contrast, when the cutoff point was < − 0.113, HT was diagnosed.

## Discussion

AITD is caused by dysregulation of the thyroid immune system. AITD is an organ-specific autoimmune disease mediated by T lymphocytes [1, 16]. AITD includes two major clinical manifestations: GD and HT, both of which are characterized by infiltration of thyroid parenchymal lymphocytes and elevation of thyroid antibodies. The clinical features of GD and HT are mainly thyrotoxicosis and hypothyroidism, respectively. Epidemiological data suggest that the interaction between genetic susceptibility and the environment is a key factor for the occurrences of GD and HT. However, the mechanism of pathogenesis is still not clear.

It is still difficult to make differential diagnosis of GD and HT. The main clinical manifestation of GD is hyperthyroidism, while the clinical features in the early stage of HT also manifested by hyperthyroidism. At present, clinicians distinguish GD and HT mainly based on clinical characteristics, thyroid antibodies, and RAIU.

Generally, TPOAb and TGAb are mainly used to diagnose HT, while TRAb is mainly used for the diagnosis of GD. About 70% of GD patients have positive TPOAb and TGAb. However, TRAb can also be significantly increased in HT patients [9]. TPOAb, TGAb, TRAb can only provide a reference for the clinical diagnosis of GD and HT. To confirm of the diagnoses, the measurement of TSAb and TSBAb is not clinically feasible despite of their accuracy. Similarly, the RAIU is increased in GD patients. Such increase in HT patients is not as high as GD patients, but there is no clearly defined range and clinicians can only judge based on clinical experience.

Therefore, for the initial onset of GD and HT, it may be difficult to differentiate, and so far there is no clear and objective diagnostic criterion in clinical practice. Without properly and effectively guide clinical treatment, patients might accept the wrong treatment plan, leading to serious adverse consequences. Therefore, in this study, the pathological results were used as the basis for the diagnosis of GD and HT.

After screening, these two groups enter model 1 and 2 respectively. The larger value of each variable in the two models indicated the higher probability to diagnose GD. This is the first time to establish a differential diagnosis model using the clinical features of GD and HT and related laboratory results.

From the perspective of area under the ROC curve of the two models, model 1 and model 2 have high diagnostic value for the identification of GD and HT. It is recommended to use − 0.596 as the cutoff point for diagnosing GD and HT when using Model 1. Similarly, it is recommended to use − 0.113 as the cutoff point to diagnose GD and HT when using model 2. If the AUC is greater than cutoff point, GD should be diagnosed. Conversely, if the AUC is less than the cutoff point, HT should be diagnosed. When the patient’s etiological analysis is not clear, our model can help clinicians make diagnosis based on readily available clinical data. Furthermore, the two models can be used to choose laboratory tests that can be performed (local hospitals usually have the ability to perform detection of thyroid antibodies).

However, there are also some shortcomings in our model. For example, model 1 is currently only applicable to hospitals that can carry out radionuclide scanning, and there are limitations for some local hospitals in China. In addition, due to the limited sample size, we have not yet verified the results for large sample sizes. Future studies will further optimize and verify the model.

## Conclusions

In the present study, clinical indexes of GD and HT including FT3, FT4, TGAb, TPOAb, TRAb, 2-h RAIU, 24-h RAIU and gender were used to establish a clinical diagnostic model, which can quantitative assist for the diagnosis of GD and HT. Therefore, it helps clinicians differentiate GD and HT, making it easier and timelier to provide patients with optimal treatments.

## Abbreviations

GD: Graves’ disease
HT: Hashimoto’s thyroiditis
RAIU: radioactive iodine uptake
ROC: receiver operating characteristic
AITD: autoimmune thyroid disease
TRAb: thyrotropin receptor antibody
TGAb: thyroglobulin antibody
TPOAb: thyroid peroxidase antibody
TSAb: thyroid stimulating antibody
TSBAb: thyroid stimulating blocking antibody
FT3: free T3
FT4: free T4
TSH: thyroid stimulating hormone
AUC: area under the curve
